# Patient engagement in quality of life research

**DOI:** 10.1007/s11136-024-03727-8

**Published:** 2024-08-03

**Authors:** Natasha Roberts, Sandra Zelinsky, Current Co-Chairs for the Patient Engagement Special Interest Group for the International Society for Quality of Life, Sadia Ahmed, Christel McMullan, Leonie Young

**Affiliations:** 1 STARS Alliance, Metro North Health and The University of Queensland, 296 Herston Rd, Herston, QLD 4029 Australia; 2 Centre for Clinical Research (UQCCR), The University of Queensland, Herston, QLD 4029 Australia; 3 Alberta SPOR SUPPORT Unit, Community Health Services Department, Cumming School of Medicine, Patient Engagement Team and University of Calgary, Calgary, Alberta Canada

The International Society of Quality of Life (ISOQOL) Patient-Engagement Special Interest Group (PE-SIG) has just celebrated its ten-year anniversary. The first PE-SIG symposium World Café meeting of 60 participants took place at the October 2013 ISOQOL Congress [1]. In October 2023, the ISOQOL PE-SIG again explored patient engagement in quality of life research. This symposium included case study presentations by patient research partners leading work in clinical trials, primary care and paediatrics. 

In preparation for the 2023 symposium, the ISOQOL PE-SIG chairs reached out to the membership of ISOQOL to seek their perspectives on patient engagement. Considerable variation in the terminology for patient research partners emerged, example titles included “patient advocates” and “consumer representatives”. Shared guiding principles for patient engagement also varied, with many resources available globally, for example Patient and Public Involvement and Engagement (PPI) in the United Kingdom. Interestingly, it was also identified that there is considerable variation in definitions for patient engagement itself. The broad response from across the ISOQOL demonstrated that the membership is involved in patient engagement activities internationally by collaborating with professional organisations, industry, academia, government and research funding bodies. It appears that there are many ways to support meaningful patient engagement in research.

The literature shows that patient engagement activities have increased exponentially since the original *PE Cafe* [2]. The considerable variation in terminology and resources may be due to these emerging in isolation from each other at the same time. Participants from the first ISOQOL PE-SIG symposium identified that there was a lack of understanding on how to engage patients in quality of life research, and whether there was evidence to support this as a beneficial exercise [1]. At that time, there was a lack of guidelines or toolkits which could be used to support patient engagement in research generally. Now these are at hand, evaluating the process of patient engagement and impact on health research outcomes is often an expectation from many funders and policy makers [2, 4]. Having the right patient partners can make a difference to the outcomes of the research, and when this is done well, the outcomes have greater impact [3]. Work published by Trisha Greenhalgh and colleagues has identified that a purposive patient engagement framework can be co-designed so that it is fit for purpose [2]. Despite this progress, the evidence base specific to quality of life research is still emerging.

The ISOQOL PE-SIG strives to ensure that the patient voice is represented in activities through the advancement of patient engagement in health-related quality of life research. It also aims to build knowledge about methods that incorporate lived experiences and knowledge, and by supporting the current and prospective patient research partner community. Having patient research partners as investigators on research teams is still not routine practice, despite an interest and willingness from researchers. It is also hoped that a better understanding of lived experience can continue to be established. Noting that it is not just understanding the experience with a given health condition, but how each person’s environment and predisposing factors influence access to and experiences with healthcare [2].

 Partnerships with patient, family, and community research partners are valued by the ISOQOL PE-SIG as they ensure scientific knowledge is relevant, and directly addresses the needs of all those requiring healthcare. Outside of annual ISOQOL congresses, the PE-SIG has hosted patient research partner “meetups” and webinars, and encouraged discussion in the Teamwork discussion board. Through such activities, the ISOQOL PE-SIG members have shared their learnings, projects, experiences, and opportunities for improvement.

However, there is much that still needs to be done to ensure inclusive measurement of quality of life and patient experiences [5]. Novel approaches are needed so that we measure what matters, and so that the results are  meaningful. Using a variety of research methods will ensure optimal involvement of patient research partners in health-related quality of life research theory and methods; and to application in clinical research, clinical practice and in policy. Mixed methods approaches can be more useful, for example, to include more diverse populations in research and healthcare. Digital storytelling was presented as a workshop at the 2021 ISOQOL Conference,  to demonstrate how visual media captures quality of life from the patients’ perspective. The key themes presented in digital stories may be  lost when traditional methods are used on their own. Sharing insights into the performance of a methodological approach when reporting any research can help build the evidence base.

It seems whilst there has been much progress since the 2013 World Café, there remain important opportunities to co-create a framework that supports rigorous evaluation of patient engagement, to “actively engage patient research partners to shape the future ISOQOL strategy” [2]. In 2023, the ISOQOL PE-SIG welcomed its second patient research partner co-chair, and aims to continue to support networks for patient research partners, industry partners, clinicians, researchers and policy makers. By taking this moment to assess the progress of the ISOQOL PE-SIG, we acknowledge the work of those in ISOQOL who have preceded us, and those into the future.
